# Disruption of MicroRNA Expression in Human Airway Cells by Diesel Exhaust Particles Is Linked to Tumorigenesis-Associated Pathways

**DOI:** 10.1289/ehp.0900756

**Published:** 2009-06-18

**Authors:** Melanie J. Jardim, Rebecca C. Fry, Ilona Jaspers, Lisa Dailey, David Diaz-Sanchez

**Affiliations:** 1 Human Studies Division, National Health and Environmental Effects Research Laboratory, U.S. Environmental Protection Agency, Chapel Hill, North Carolina, USA; 2 Environmental Sciences and Engineering, Gillings School of Global Public Health; 3 Center for Environmental Medicine, Asthma, and Lung Biology and; 4 Department of Pediatrics, School of Medicine, University of North Carolina, Chapel Hill, North Carolina, USA

**Keywords:** air–liquid interface, DEP, diesel, gene regulation, human bronchial epithelial cells, microRNA, tumorigenesis

## Abstract

**Background:**

Particulate matter (PM) is associated with adverse airway health effects; however, the underlying mechanism in disease initiation is still largely unknown. Recently, microRNAs (miRNAs; small noncoding RNAs) have been suggested to be important in maintaining the lung in a disease-free state through regulation of gene expression. Although many studies have shown aberrant miRNA expression patterns in diseased versus healthy tissue, little is known regarding whether environmental agents can induce such changes.

**Objectives:**

We used diesel exhaust particles (DEP), the largest source of emitted airborne PM, to investigate pollutant-induced changes in miRNA expression in airway epithelial cells. We hypothesized that DEP exposure can lead to disruption of normal miRNA expression patterns, representing a plausible novel mechanism through which DEP can mediate disease initiation.

**Methods:**

Human bronchial epithelial cells were grown at air–liquid interface until they reached mucociliary differentiation. After treating the cells with 10 μg/cm^2^ DEP for 24 hr, we analyzed total RNA for miRNA expression using microarray profile analysis and quantitative real-time polymerase chain reaction.

**Results:**

DEP exposure changed the miRNA expression profile in human airway epithelial cells. Specifically, 197 of 313 detectable miRNAs (62.9%) were either up-regulated or down-regulated by 1.5-fold. Molecular network analysis of putative targets of the 12 most altered miRNAs indicated that DEP exposure is associated with inflammatory responses pathways and a strong tumorigenic disease signature.

**Conclusions:**

Alteration of miRNA expression profiles by environmental pollutants such as DEP can modify cellular processes by regulation of gene expression, which may lead to disease pathogenesis.

Morbidity and mortality attributable to air pollution continues to be a growing problem worldwide (Brunekreef and Holgate 2002). Both epidemiologic and clinical studies have demonstrated a strong link between exposure to particulate matter (PM) and detrimental health outcomes (Diaz-Sanchez and Riedl 2005; McCreanor et al. 2007; Riedl and Diaz-Sanchez 2005). Diesel exhaust particles (DEP) are the largest single source of vehicular-emitted airborne PM and can persist in the air, where they are readily inhaled and deposited throughout the respiratory tract (Cao et al. 2007b). DEP-induced toxicity is attributed mainly to its chemical composition, consisting of a carbonaceous core to which organic and inorganic species can become adsorbed. Diesel exhaust has been classified by the U.S. Environmental Protection Agency (EPA) as a likely carcinogen, due primarily to epidemiology studies that have shown its association with lung cancer (Ris 2007; U.S. EPA 2002). Additionally, exposure to DEP has been linked with several adverse respiratory health events, including pulmonary inflammation, increased susceptibility to respiratory infections, increased risk of cancer, and exacerbation of asthma and chronic obstructive pulmonary diseases. Despite the wealth of studies on health effects, the underlying molecular mechanisms by which DEP initiates disease still remain elusive (Diaz-Sanchez 1997; Diaz-Sanchez et al. 1994, 2000a, 2000b; Garshick et al. 2008; Holder et al. 2008; Lewtas 2007; Moller et al. 2008).

MicroRNAs (miRNAs) constitute a class of small, evolutionary conserved, non-coding RNAs critical for regulation of gene expression. miRNAs are single-stranded and 19–24 nucleotides in length, and have been found to be potent regulators of several cellular processes, including apoptosis, proliferation, and differentiation (Flynt and Lai 2008; Roush and Slack 2008; Singh et al. 2008). Unlike other small RNAs, such as siRNAs, miRNAs are transcriptionally regulated by RNA polymerase II and are the resultant product of a larger pri-miRNA strand that contains a 5′ cap and poly(A) tail (Kim 2005). Mammalian miRNAs imperfectly base pair with the 3′ untranslated region (UTR) of target mRNAs, ultimately leading to target degradation or inhibition of protein translation (Pillai et al. 2007). The emerging role of miRNAs as regulators of a wide spectrum of biological functions suggests that they play a pivotal role in protecting organ tissues from diseases. Aberrant miRNA expression has been implicated in the pathogenesis of several human diseases, such as solid tissue and hematologic malignancies, heart disease, congenital organ defects, and neurodegenerative diseases (Nana-Sinkam et al. 2009; Schickel et al. 2008; Singh et al. 2008). Despite the clear importance of miRNAs in several biological functions and the discovery of > 500 identified miRNA sequences, little is understood regarding their regulation. In recent years, modulation of miRNA repression has been shown to be a highly dynamic process, as evidenced by studies showing the rapid alleviation of miRNA-mediated translational repression in response to specific cellular needs (Bhattacharyya et al. 2006; Liu et al. 2005).

Although the study of miRNAs in disease is still in its infancy, it has become increasingly apparent that miRNAs play a pivotal role both in lung development and in maintaining the lung in a disease-free state (Nana-Sinkam et al. 2009; Schickel et al. 2008). For example, miRNA knockout studies have shown that loss of function of the *miR-17-92* cluster leads to hypoplastic lung (Ventura et al. 2008). Comparison of normal lung tissues with those from different lung cancers reveals significant differences in miRNA profiles and has shown that different miRNAs can act both as tumor suppressors and oncogenes (Yanaihara et al. 2006; Zhang et al. 2007). Indeed, miRNA profiles have been proposed as a diagnostic tool to predict survival and relapse in lung cancer patients (Yu et al. 2008). Although these data suggest that miRNA regulation may be amenable to environmental insults, very few studies have focused on studying how miRNA profiles are altered by exogenous stimuli.

We hypothesized that exposure to DEP can alter miRNA-regulated gene expression in human airway epithelial cells, one of the first targets of PM inhalation. In this initial study, we tested this hypothesis by analyzing the expression pattern of miRNAs in differentiated human bronchial cells (HBEC) grown at air–liquid interface (ALI), an *in vitro* cell culture system that more accurately mimics the structural and functional characteristics of the airway epithelium found *in vivo* than does a standard submerged culture (Ross et al. 2007). We show that miRNA expression profiles can change in response to DEP and that these changes may affect the underlying molecular mechanisms controlling human airway diseases.

## Materials and Methods

### Cell culture

Primary human bronchial epithelial cells were obtained from a healthy, nonsmoking adult donor. The protocol and consent form were approved by the University of North Carolina School of Medicine Committee on the Protection of the Rights of Human Subjects. Participation of human subjects in this study did not occur until after informed written consent was obtained. Cells were obtained by cytologic brushing at bronchoscopy and expanded to passage two in bronchial epithelial growth media (Clonetics, Walkersville, MD). Cells were then plated onto collagen-coated filter supports with a 0.4 μm pore size (Trans-CLR; Costar, Cambridge, MA) and cultured in a 1:1 mixture of bronchial epithelial cell basic medium and Dulbecco modified Eagle medium-HEPES (DMEM-H) with SingleQuot supplements (Cambrex, Walkersville, MD), bovine pituitary extracts (13 mg/mL), bovine serum albumin (1.5 μg/mL), and nystatin (20 U). Upon confluence, all-*trans*-retinoic acid was added to the medium, and ALI culture conditions (removal of the apical medium) were created to promote differentiation. Mucociliary differentiation was achieved 21 days later.

### Treatment with suspended DEP

DEP were generated from a light-duty four-cylinder diesel engine (4JB1 type, Isuzu automobile; Isuzu Motors, Tokyo, Japan) using standard fuel and generously provided by M. Sagai (National Insitute for Environmental Studies, Tokyo, Japan). DEP (10 μg/cm^2^) were freshly prepared and suspended in a total of 200 μL media by sonication. Suspended particles were then added to the apical side for 2 hr and then removed to reestablish ALI. Control cells received 200 μL fresh media for 2 hr to exclude potential changes in miRNA expression due to liquid exposure. Media was then removed to reestablish ALI. We found no indications of cytotoxicity upon treatment with DEP. Total RNA was isolated 24 hr after treatment.

### Microarray analysis

Total RNA was isolated with Trizol (Invitrogen, Carlsbad, CA) according to the manufacturer’s protocol. miRNA arrays (Agilent Technologies, Santa Clara, CA) contained 723 human miRNAs. miRNA sequences were obtained from the Sanger miRBase, Release 10.1. (miRBase 2009). We performed labeling and hybridization according to the manufacturer’s protocol. The same sample was run once at 100 ng and once at 400 ng of input RNA to reduce the number of false positives and false negatives. Overall signal intensity was low, even in the 400-ng sample. Microarray results were extracted using Agilent Feature Extraction Software (Agilent Technologies) and analyzed using Spotfire (TIBCO, Somerville, MA). To obtain a profile of miRNAs that were significantly induced or repressed by DEP, we set a threshold of 1.5-fold. Targets for miRNAs that were changed by a minimum of 4-fold were identified using the online databases miRDB (2009) and TargetScan (2009). Only targets with a score of > 80 or < −0.50 were considered for analysis. Molecular network analysis was carried out using the Ingenuity Knowledge Base, “a repository of molecular interactions, regulatory events, gene-to- phenotype associations, and chemical knowledge” (Ingenuity Systems 2009). We generated molecular networks using a data set containing gene identifiers derived from a list of putative gene targets for each miRNA studied. These genes were overlaid onto a global molecular network developed from information contained in the Ingenuity Pathways Knowledge Base. Networks of these focus genes were then algorithmically generated based on their connectivity. The functional analysis of a network identifies the biological functions and/or diseases that are most significant to the genes in the network. The network genes associated with biological functions and/or diseases in the Ingenuity Pathways Knowledge Base were considered for the analysis. A Fisher’s exact test was used to calculate a *p*-value determining the probability that each biological function and/or disease assigned to that network is due to chance alone.

### Quantitative real-time polymerase chain reaction (qRT-PCR)

We isolated total RNA with Trizol (Invitrogen, Carlsbad, CA) according to the manufacturer’s protocol. cDNA was synthesized using the TaqMan MicroRNA Reverse Transcription Kit (Applied Biosystems, Foster City, CA). Predeveloped oligonucleotide primer and probe sets were purchased from Applied Biosystems (TaqMan miRNA assays). We performed quantitative fluorogenic amplification of cDNA using the ABI Prism 7500 Sequence Detection System (Applied Biosystems), primers and probes of interest, and TaqMan Universal PCR Master Mix (Applied Biosystems). The relative abundance of each miRNA was calculated using the 2^−ΔΔC_T_^ method (Litvak and Schmittgen 2001). U6 small nuclear RNA was used as an internal control for normalization. Independent samples were run in triplicate and values are expressed as mean ± SD.

## Results

### DEP alters miRNA expression in human airway epithelial cells

**Figure 1:**
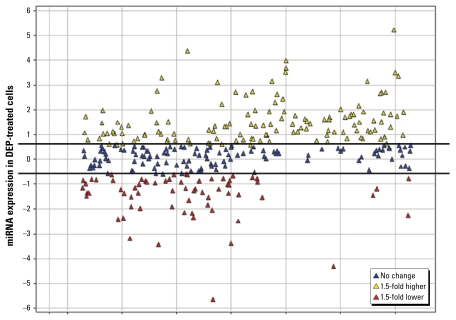
Scatterplot showing the relationship between the expression of miRNAs in DEP-treated cells (10 μg/cm^2^ DEP) compared with control cells. Black lines indicate the 1.5-fold threshold for control cells. All values are expressed in log2.

In this study, we set out to identify novel molecular mechanisms of DEP-induced adverse health effects. Figure 1 shows that DEP treatment causes differential expression patterns of miRNAs in differentiated human bronchial epithelial cells. We observed 264 miRNAs present in control cells, representing a baseline miRNA expression profile. In contrast, cells stimulated with a low concentration of DEP had 293 detectable miRNAs. A total of 313 human miRNAs were identified as being expressed in either control or DEP-treated samples with an RNA input of 400 ng. Furthermore, 130 miRNAs showed an increase of ≥ 1.5-fold in response to DEP, whereas 67 showed a decrease ≥ 1.5-fold in expression [summarized in Table 1; see also Supplementary Material, Table 1, available online (doi:10.1289/ehp.0900756.S1 via http://dx.doi.org/)]. The expression of 116 miRNAs did not significantly change.

To identify relevant miRNAs regulated in response to DEP, we elected to apply a more restrictive set of criteria for further data analysis. As seen in Table 2, the expression of 12 miRNAs was found to be increased or decreased by at least 4-fold in the 400-ng samples, and increased or decreased by at least 1.5-fold in the 100-ng samples

### Validation of miRNA expression

**Figure 2:**
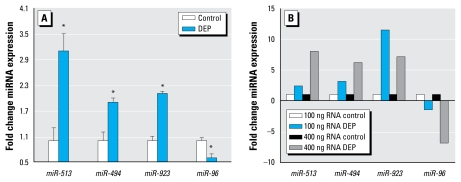
Validation of miRNA expression. (*A*) qRT-PCR for miRNA expression in control versus DEP-treated cells. (*B*) MiRNA expression values as ratio of DEP to control in both when 100 ng total RNA and 400 ng total RNA were used as inputs. *p < 0.05.

To validate our array data, we selected four miRNAs significantly changed by DEP and performed qRT-PCR to validate array observations. These miRNAs were chosen for further analysis because of their overall expression intensities as observed on the array. In agreement with our microarray data, each miRNA showed the same trend in regulation, specifically, *miR-513a-5p*, *miR-494*, and *miR-923* each increased in response to DEP, whereas *miR-96* decreased in DEP-treated samples (Figure 2). As observed in the array data, *miR-513a-5p* was the greatest induced miRNA, with a relative 3-fold increase over control cells, whereas miR-96 expression was reduced by half.

### Network analysis of miRNAs highly modulated by DEP

**Figure 3:**
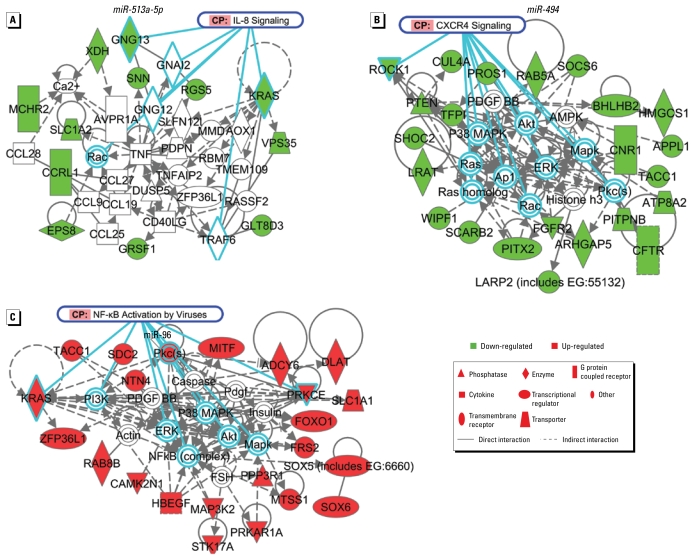
Subnetworks of select putative DEP-regulated gene transcripts show an enrichment of inflammatory response. Gene networks displaying putative interactions using potential gene targets of (*A*) *miR-513a-5p* (*p* < 10^−27^), (*B*) *miR-494* (*p* < 10^−49^), and (*C*) *miR-96* (*p* < 10^−49^). Solid-colored shapes indicate molecules identified as putative targets for each respective miRNA. Green indicates putative transcripts that are repressed, and red indicates putative gene targets that may be up-regulated; pathway enrichment is highlighted in blue.

To determine the biological relevance of the identified miRNAs, networks were mapped for each of the putative input miRNA targets for three significantly altered miRNAs (Figure 3). We assessed the potential biological functions of select miRNAs by identifying putative miRNA targets using TargetScan and miRDB for *miR-513a-5p*, *miR-494*, *miR-96*, and *miR-923*. Targets were chosen based on a reported context score of ≥ 80 (miRDB) or a score of ≤ −0.5 (TargetScan); neither program generated target mRNAs that fit our criteria for *miR-923*, so it was excluded from any further analysis. Identified molecular networks were highly enriched for inflammatory responses that correlated well with canonical signaling pathways, such as interleukin (IL)-8, nuclear factor kappa B (NF-κB), and chemokine (C-X-C motif) receptor 4, where these targets could be acting.

**Figure 4:**
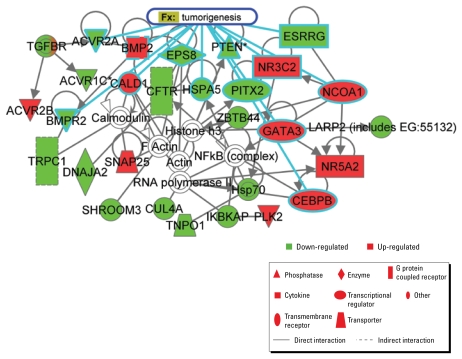
Identification of candidate molecular network for miRNAs most highly changed in response to DEP. We analyzed putative targets from miRNAs in Table 2 for significant pathway enrichment of molecular interactions and identified a significant interactome containing putative DEP-modulated gene products involved in tumorigenesis (highlighted in blue). Red represents putative DEP-induced transcripts, and green represents putative DEP-repressed transcripts; pathway enrichment is highlighted in blue; *p* < 10^−47^.

To further analyze the putative effects of differential miRNA expression profiles in DEP-exposed ALI cells, we again used TargetScan and miRDB to identify possible mRNA targets, with the same scoring criteria used above, for the remaining miRNAs listed in Table 2. These targets were then analyzed for enriched molecular networks. As such, we identified a total of 27 networks, the highest ranked of which was used for further examination (Figure 4). To better understand the potential health effects of the observed miRNA expression profile of DEP-exposed cells, we focused our analysis on putative disease signatures and functional relationships. Interestingly, the highest-ranked identified network was highly enriched for cancer, with an emphasis on tumorigenic processes.

## Discussion

This study is the first demonstration that exposure to ambient air pollutants such as DEP can disrupt miRNA expression patterns in human airway cells, and that this may lead to gene expression changes associated with disease initiation. Using an *in vitro* model system of the human lower respiratory tract, we show that DEP exposure alters miRNA expression. Given the number of miRNAs whose expression pattern changed in response to DEP, we believe that regulation of gene expression by miRNAs represents a novel and likely mechanism through which DEP can disrupt cellular homeostasis, leading to the development of a pathologic disease state.

To assess potential biological mechanisms of acute DEP exposure, we identified putative targets for *miR-513a-5p*, *miR-494*, and *miR-96*, three miRNAs whose expression was significantly altered after DEP exposure. Based on these putative targets, we were able to map candidate signaling networks to identify any potential network commonalities between the identified targets for each miRNA. Furthermore, although each miRNA had a different set of putative targets, it was evident that these putative targets modulate the DEP-induced inflammatory response. Although the ability of DEP to cause release of several proinflammatory cytokines such as IL-8, IL-6, and tumor necrosis factor-α has been reported in population-based and experimentally based studies (Cao et al. 2007a, 2007b; Diaz-Sanchez 1997; Diaz-Sanchez et al. 1994, 2000a, 2000b; Finkelman et al. 2004; Holder et al. 2008), the notion that these effects and the resolution of these events may be due to changes in the expression pattern of miRNAs has not yet been shown and is an area for further exploration. Interestingly, our analysis suggested that inflammatory pathways are potentially regulated by increasing expression of some miRNAs while decreasing the expression of others, suggesting that miRNA response to exogenous insults involves an intricate web of gene networks that must be kept in balance for eliciting and modulating cellular responses.

Very little is known about *miR-494*; however, its putative target mRNAs indicate an enriched gene network for NF-κB canonical and virus-activated signaling. In line with these results, it has been shown previously that in addition to increasing susceptibility to influenza virus, DEP can enhance viral attachment and entry into human airway epithelial cells (Ciencewicki et al. 2006; Jaspers et al. 2005). It has been shown recently that *miR-513* plays a role in regulating interferon-γ (IFN-γ)-induced apoptosis. In response to the proinflammatory cytokine IFN-γ, *miR-513* expression levels decrease and B7-H1 protein levels increase, subsequently leading to cell death (Gong et al. 2009). *miRNA-513* was found to target the 3′ UTR of B7-H1, resulting in translational repression and ultimately leading to an inhibition of apoptosis when *miR-513* was overexpressed (Gong et al. 2009). Although these studies were conducted in a different cell model from our study, these experimentally based findings support our computer-generated network analysis in that both types of analysis suggest that *miR-513* family members may be participating in inflammatory signaling pathways. Overall, gene network analysis on putative targets for *miR-494* and *miR-513* lead us to speculate that these two miRNAs may play a role in the regulation of a cell’s inflammatory response. Whether these miRNAs are involved in inducing inflammation or in the resolution or fine-tuning of the signal is yet to be determined.

Although most miRNAs do not have any known, biologically confirmed targets, *miR-96* has been shown to regulate levels of the protein arginine methyltransferase (PRMT5) (Pal et al. 2007). PRMT5 interacts with human SWI/SNF and methylates histones H3R8 and H4R3, ultimately suppressing gene expression through chromatin modifications. Indeed, PRMT5 is highly expressed in some cancer cell lines and when knocked down, interferes with cell growth, indicating that it may play a crucial role in silencing tumor suppressors (Pal et al. 2007). Cao et al. (2007a) showed that DEP induces chromatin modifications leading to up-regulation of the Cox-2 gene after 4 hr of treatment. Although speculative at this point, it is possible that if PRMT5 is indeed targeted by *miR-96* in human bronchial epithelial cells, our observation that *miR-96* levels decrease 24 hr after DEP treatment may represent signal resolution and subsequent silencing of some genes.

Although DEP are classified as potential causal carcinogenic agents, the mechanism by which they alter cellular function remains largely unknown (Lewtas 2007; Moller et al. 2008; Schins and Knaapen 2007). Several studies have documented the mutagenic potential of different DEP and their chemical constituents; however, a link between these data and relevant biological consequences has not been established. Most of these studies used different strains of *Salmonella typhimurium* to show that DEP can induce frameshift mutations and base substitutions, presumably suggesting that DEP have a high tumorigenic potency (DeMarini et al. 2004). However, no one has yet shown that these two phenotypes are directly linked, specifically in normal human airway epithelial cells.

There are different types of DEP, and although the magnitude of effects varies depending on inherent chemical compositions, they have all been found to possess mutagenic potential (DeMarini et al. 2004). The effects of specific chemicals on particular miRNA expression patterns are beyond the scope of this study, but is an important question that we are currently exploring. Animal models have shown that different DEP samples and their chemical components can induce pulmonary toxicity in mice (Hashimoto et al. 2007; Schins and Knaapen 2007; Singh et al. 2004). Induction of oxidative stress and the formation of bulky DNA adducts—most likely due to different DEP constituents—observed in these animals are thought to be the major mechanism driving carcinogenesis. Several groups have shown a positive relationship between DEP exposure and lung tumor formation in rodents; however, a common criticism of these data is that tumor burden can be attributable to particle overload (Schins and Knaapen 2007). Indeed, other data suggest that the carbonaceous core of DEPs alone can induce tumor formation, further supporting the notion that these animals might have been exposed to very high dosages of particles (Gallagher et al. 1994).

When considering putative targets for all significantly up-regulated and down-regulated miRNAs, gene network analysis revealed a complex molecular interactome enriched for cancer-associated genes. It has been well established that miRNAs play a pivotal role in tumorigenesis (He et al. 2005; Johnson et al. 2005; Lu et al. 2007; Ventura et al. 2008) and can be used as tumor classifiers (Gaur et al. 2007; Lu et al. 2005; Yu et al. 2008). Although current data indicate that DEP are likely carcinogens, it will be interesting to see in future studies whether aberrant DEP-induced miRNA expression changes can be functionally linked to tumorigenesis.

Very few studies have shown that exposure to environmental pollutants can alter miRNA expression profiles. Mice exposed to RDX (hexahydro-1,3,5-trinitro-1,3,5-triazine), an environmental contaminant, had severely altered miRNA profiles in liver and brain, two target organs for induced health effects (Zhang and Pan 2009). In addition, Izzotti et al. (2008) recently demonstrated that cigarette smoke significantly alters miRNA profile in the lung tissue of exposed rats. These studies strongly suggest that miRNA expression can greatly change with chronic exposure to toxicants, and it will be interesting to see if these results can be directly linked to a disease outcome. It would also be interesting to see how similar the miRNA profiles in individual cell types are within each target organ, both at baseline and after exposure.

We chose to focus our study on acute exposure in a single human cell type, which allowed us to show that miRNA expression can change in response to an environmental insult. Additionally, our study shows that miRNA expression can change in the absence of a disease or a predisease state that likely already contains permanent miRNA expression changes within a tissue, thus allowing our model system of acute exposure to be used to tease apart molecular mechanisms of miRNA regulation of gene expression in future studies. To gain a better understanding of the potential molecular responses, we generated biological networks using predicted targets of those miRNAs that significantly changed response to treatment with DEP. Although specific miRNA-target interactions remain largely unknown, the enrichment of predicted target genes sharing a common biological network suggests that miRNAs may regulate gene expression in a somewhat function- or network-specific mechanism. Because miRNAs may target > 100 mRNAs and a single mRNA may be targeted by several miRNAs (Flynt and Lai 2008), it is not surprising that miRNAs can act in several networks and in different capacities to regulate a wide range of biological functions. It will be particularly interesting to see how the up-regulated and down-regulated miRNAs act together to govern the cellular response to DEP in bronchial epithelial cells. Given the complexity of miRNA regulation and the current lack of validated targets, further in-depth studies are necessary to fully understand the functional role of miRNAs in regulating cellular responses to exogenous stimuli and in their contribution to human disease.

## Conclusions

Our results clearly demonstrate the impact of DEP exposure on the expression of miRNAs in differentiated human bronchial epithelial cells. The observed DEP-induced changes in miRNA may have the potential to contribute to adverse health effects by directly or indirectly altering cellular homeostasis. Further studies on DEP-induced miRNA profile changes and subsequent identification of mRNA targets will allow for a more comprehensive study of DEP-induced pathogenic states and underlying molecular mechanisms of disease. Environmental modulation of gene expression is currently the focus of intense studies, as many questions regarding toxic substance exposure and human health outcomes remain unanswered. Overall, future studies on miRNA response profiles may contribute to the development of disease- and exposure-specific biomarkers for diesel exhaust and for other environmental toxins to better monitor associated adverse health outcomes.

## Figures and Tables

**Table 1:** Summary of array results.

Sample RNA concentration (ng)	miRNAs detected (*n*)	≥ 1.5-Fold increase (*n*)	≥ 1.5-Fold decrease (*n*)	≥ 2-Fold increase (*n*)	≥ 2-Fold decrease (*n*)
100	210	41	38	14	10
400	313	130	67	94	42

**Table 2:** miRNAs induced or repressed ≥ 4-fold in cells exposed to 10 μg/cm^2^ DEP.

miRNA	DEP/control ratio	Clustered miRNAs	Function
*miR-513c*	16.0	*miR-513b*	Unknown
*miR-513b*	13.0	*miR-513c*	Unknown
*miR-513a-5p*	11.3	None	Represses IFN-γ–induced apoptosis (Gong et al. 2009)
*miR-923*	10.6	None	Unknown
*miR-494*	9.2	miRs 379,411,299, 380,1197, 323,758, 329-1,329-2,543,496	Highly expressed in retinoblastoma (Zhao et al. 2009)
*miR-338-5p*	4.6	*miR-1250*, *miR-657*	Axonal regulation of local cytochrome c oxidase IV mRNA levels in axons (Aschrafi et al. 2008)
miR-31*	0.23	None	Decreased in ischemic retina; up-regulated in colorectal tumors; levels predict colorectal cancer tumor stage (Bandres et al. 2006; Shen et al. 2008; Slaby et al. 2007)
*miR-26b*	0.22	None	Decreases proapoptotic signaling in hypoxic environment; down-regulated in lungs of rats exposed to cigarette smoke (Izzotti et al. 2008; Kulshreshtha et al. 2007)
*miR-96*	0.20	*miR-183, miR-182*	Modulates methylation of H3R8 and H4R3 via regulation of PRMT5 protein levels; retinal disease models (Loscher et al. 2007, 2008; Pal et al. 2007)
*miR-27a*	0.20	*miR-23a, miR-24-2*	Regulation of fat metabolism and cell proliferation; megakaryocytic differentiation; oncogenic in several tissues; regulation of drug resistance (Ben-Ami et al. 2009; Ji et al. 2009; Zhu et al. 2008)
*miR-135b*	0.19	None	Unknown
*miR-374a*	0.18	*miR-545*	Unknown

INF-γ, interferon-γ.
